# Assessment of Vitamin Status in Patients with Nontuberculous Mycobacterial Pulmonary Disease: Potential Role of Vitamin A as a Risk Factor

**DOI:** 10.3390/nu11020343

**Published:** 2019-02-05

**Authors:** Jongwon Oh, Hyung-Doo Park, Su-Young Kim, Won-Jung Koh, Soo-Youn Lee

**Affiliations:** 1 Department of Laboratory Medicine and Genetics, Samsung Medical Center, Sungkyunkwan University School of Medicine, 81 Irwon-ro, Gangnam-gu, Seoul 06351, Korea; tltc@naver.com (J.O.); nayadoo@hanmail.net (H.-D.P.); 2 Division of Pulmonary and Critical Care Medicine, Department of Medicine, Samsung Medical Center, Sungkyunkwan University School of Medicine, 81 Irwon-ro, Gangnam-gu, Seoul 06351, Korea; suyoung5505@hanmail.net; 3 Department of Clinical Pharmacology & Therapeutics, Samsung Medical Center, Seoul 06351, Korea

**Keywords:** nontuberculous mycobacteria, nutrition, vitamin

## Abstract

As microbiological diagnostic techniques improve and the frequency of nontuberculous mycobacterial pulmonary disease (NTM-PD) infection increases worldwide, NTM-PD is becoming increasingly important to clinicians and researchers. Vitamin activity has been associated with the host immune response in tuberculosis; however, such information is very limited in NTM-PD. We performed a case-control study in 150 patients with NTM-PD and 150 healthy controls to investigate serum vitamin status. We measured concentrations of vitamins A, D, and E along with homocysteine and methylmalonic acid (MMA) as indicators of vitamin B_12_ deficiency, using high-performance liquid chromatography (HPLC) or HPLC-tandem mass spectrometry. The serum concentrations of vitamins A and E were significantly lower in patients with NTM-PD than in healthy controls (1.5 vs. 2.1 µmol/L, *p* < 0.01 for vitamin A; and 27.3 vs. 33.1 µmol/L, *p* < 0.01 for vitamin E). In contrast, the serum concentrations of vitamin D and homocysteine were not significantly different between the two groups. Vitamin A deficiency (< 1.05 µmol/L) was significantly more prevalent in patients with NTM-PD than in healthy controls (*p* < 0.01) and was associated with an 11-fold increase in risk of NTM-PD. Multiple vitamin deficiencies were only observed in patients with NTM-PD (7.3% of all NTM-PD patients). Positive correlations were observed among vitamins (vitamins A and D; r = 0.200, *p* < 0.05; vitamins D and E, r = 0.238, *p* < 0.05; vitamins A and E, r = 0.352, *p* < 0.05). Serum vitamin status, demographic variables, and biochemical indicators were not associated with treatment outcomes. Vitamin A deficiency was strongly associated with patients with NTM-PD. Our study suggests that altered vitamin status is associated with mycobacterial disease. Future well-designed prospective studies with large patient cohorts addressing these issues are needed to clarify the significance of vitamins in NTM-PD.

## 1. Introduction 

The incidence and prevalence of nontuberculous mycobacterial pulmonary disease (NTM-PD) are increasing worldwide, as those of tuberculosis (TB) are decreasing [1,2,3]. Among NTM species, the most frequent human pathogen in many countries is *Mycobacterium avium* complex (MAC), followed by *M. abscessus* (MAB) [1,2]. MAC mainly consists of *M. avium* and *M. intracellulare*, and MAB is predominantly composed of *M. abscessus* subspecies *abscessus* (hereafter referred to as *M. abscessus*) and *M. abscessus* subspecies *massiliense* (hereafter referred to as *M. massiliense*) [4,5]. In the past, NTM-PD cases were relatively neglected compared to those of pulmonary TB. However, as microbiological diagnostic techniques improve and the frequency of NTM infection increases worldwide, NTM-PD is becoming increasingly important to clinicians and researchers [1,2].

Associations between vitamin status and host immune response to TB have been reported. For example, we previously reported that serum concentrations of vitamin A, vitamin D, and vitamin E in patients with TB were significantly lower than in healthy controls [6]. Also, Pasaki et al. reported that severe TB is associated with vitamin A deficiency [7], and Aibana et al. showed that vitamin A deficiency and low concentrations of vitamin E are associated with increased risk of TB [8,9]. Similarly, other studies have shown concentrations of vitamin E to be lower in patients with TB compared to healthy controls [6,10,11]. Vitamin D has been the most widely investigated vitamin in relation to TB, and patients with TB have lower concentrations of vitamin D than healthy subjects [12,13,14,15,16,17,18]. Additionally, a likely role of vitamin B_12_ metabolism in pathogenesis of TB was suggested by Gopinath et al. and Young et al. [19,20]. Although studies on vitamin status and pulmonary TB are numerous, there has not yet been a study to evaluate reliable biomarkers for vitamin status in patients with NTM-PD. Considering the clinical significance and increasing incidence of NTM-PD, a study investigating the association between nutritional status and NTM-PD was needed. Thus, this study aimed to investigate vitamin status in patients with NTM-PD and various parameters associated with vitamin status as well as the relationship between vitamin status and NTM treatment outcome.

## 2. Subjects and Methods

### 2.1. Study Design, Diagnosis, and Definitions

We performed a case-control study in 150 patients with NTM-PD and 150 healthy controls to investigate serum vitamin status. The study was approved by the Institutional Review Board of Samsung Medical Center (IRB No: SMC-2008-09-016). Patients with NTM-PD were recruited consecutively from April 2014 to January 2017, and the subjects provided written consent for participation in this study. All patients satisfied the diagnostic criteria for NTM-PD described in the American Thoracic Society/Infectious Diseases Society of America statement [21]. The exclusion criteria were as follows: (a) patients with cancers; (b) patients who tested positive for human immunodeficiency virus; (c) patients with hepatic or renal impairment (total bilirubin > 2.5 mg/dL, aspartate aminotransferase (AST) or alanine aminotransferase (ALT) > 3 times the upper limits of the reference range, alkaline phosphatase > 5 times the upper limits of the reference range, serum creatinine > 1.8mg/dL); (d) patients with uncontrolled bleeding disorders; (e) patients with life-threatening disease; and (f) patients with concurrent NTM infection and pulmonary TB. We used the AdvanSure Mycobacteria GenoBlot assay (LG Life Science, Seoul, Korea) to identify NTM species [22,23,24]. Patients enrolled in this study had NTM-PD caused by four major pathogens: *M. avium*, *M. intracellulare*, *M. abscessus*, and *M. massiliense*. Control groups without current or prior diagnosis of NTM-PD were randomly selected from healthy individuals who visited a health promotion center for regular health checkups. We obtained demographic data from electronic medical records, and cases and controls were individually matched for age and sex. We defined a body mass index (BMI) less than 18.5 kg/m^2^ as underweight [25]. The status of vitamin A, vitamin D, vitamin E, homocysteine and MMA (as a vitamin B_12_ status indicator) was compared between patients with NTM-PD and healthy controls. In addition, serum vitamin concentrations were also measured after treatment in patients with NTM-PD to confirm correlation with treatment outcome. The treatment outcome of NTM-PD was assessed at 12 months after antibiotic treatment initiation. Treatment success was defined as culture conversion with three consecutive negative sputum cultures and maintenance of negative culture status until the end of treatment [26].

### 2.2. Analytical Procedures

Blood samples were collected before the start of treatment during the first visit, with the patient in the fasting state. Vitamin A and E concentrations were determined using an Agilent 1200 series high-performance liquid chromatography (HPLC) system (Agilent Technologies, Waldbronn, Germany) using commercial reagent kits (Chromsystems Instruments & Chemicals GmbH, München, Germany). An Agilent 1260 Infinity LC (Agilent Technologies) coupled to an Agilent 6460 Triple Quadrupole mass spectrometer (MS) was used for measurement of vitamin D concentration. We measured homocysteine and MMA concentrations using a Xevo TQ-S tandem MS (Waters Corporation, Milford, MA, USA) equipped with an Acquity UPLC system (Waters Corporation). All assays showed good repeatability, with all the coefficients of variation below 10%. We routinely participate in external quality assurance programs such as the Proficiency Testing/Quality Management program of the College of American Pathologists (CAP) survey and the Vitamin D External Quality Assessment Scheme (DEQAS) to verify the accuracy of our assay. Serum chemistry parameters of albumin, C-reactive protein (CRP), total protein, total cholesterol, AST, and ALT were measured for assessing biochemical status by a Roche modular analyzer (Roche Diagnostics Corp., Indianapolis, IN, USA).

The vitamin deficiency status groups were defined as previously described, i.e., serum vitamin A < 1.05 µmol/L, vitamin D deficiency as a serum 25(OH)D < 20 ng/mL, vitamin E < 11.6 µmol/L, and vitamin B_12_ deficiency as a homocysteine concentration > 15 µmol/L plus an MMA concentration > 300 nmol/L [6,27,28].

### 2.3. Statistical Analysis

Continuous variables were presented as median and interquartile range (IQR). *P* values less than 0.05 were regarded as statistically significant. The assessment of normality was conducted by the Shapiro-Wilk test. We used the Wilcoxon Mann-Whitney test for continuous variables to determine the significance of differences. We calculated proportions for categorical variables, and the Chi-square test and Fisher’s exact test were used to assess equality of proportions as appropriate. The Kruskal-Wallis rank sum test with Bonferroni’s post hoc test was performed to evaluate the significance of differences in serum vitamin concentrations by etiology in NTM-PD. To investigate the associations among vitamin status, demographic data, and biochemical results, Spearman’s correlations were used. We performed logistic regression analysis on the factors associated with NTM-PD, and we conducted multivariable linear regression analysis to examine factors related to serum vitamin concentrations. These analyses were performed using IBM SPSS software v24.0 (IBM Corp., Armonk, NY, USA), MedCalc v11.5.1.0 (MedCalc Software, Mariakerke, Belgium), and SAS version 9.4 (SAS Institute, Cary, NC, USA).

## 3. Results

### 3.1. General Characteristics of the Study Population

This study comprised 150 patients with NTM-PD and 150 healthy controls, with 44 men and 106 women in each group. Of the 150 patients with NTM-PD, 64 (42.7%) had sputum specimens that were smear-positive for acid-fast bacilli. On chest radiograph, 52 (34.7%) patients had cavitary lesion(s). The etiologic organisms were *M. intracellulare* (35.3%), followed by *M. avium* (34.0%), *M. massiliense* (18.7%), and *M. abscessus* (12.0%).

The general characteristics of the patients with NTM-PD and healthy controls are summarized in Table 1. Patients with NTM-PD were more frequently underweight than were healthy controls (*p* < 0.001). The median concentrations of total protein, albumin, and CRP were higher in patients with NTM-PD than in healthy controls, whereas total cholesterol concentrations were significantly lower in the NTM-PD patients than in healthy controls.

### 3.2. Vitamin Status in the Study Population

**Figure 1:**
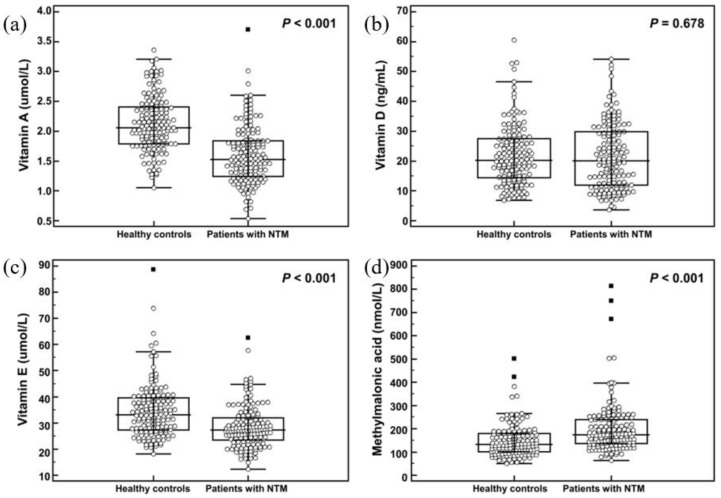
Comparison of vitamins and vitamin B_12_ status indicator between healthy controls and patients with nontuberculous mycobacteria (NTM). (**a**) vitamin A. (**b**) vitamin D (**c**) vitamin E and (**d**) methylmalonic acid

The serum vitamin concentrations of the study populations and the vitamin deficiency status groups, defined as described above, are shown in Table 2 and Figure 1. Serum concentrations of vitamins A and E in patients with NTM-PD were significantly lower than in healthy controls (*p* < 0.001). Serum MMA concentration was higher in patients with NTM-PD than in healthy controls (*p* < 0.001). However, serum concentrations of vitamin D and homocysteine were not significantly different between the two groups. In the analysis of vitamin deficiency status, vitamin A deficiency was significantly more prevalent in patients with NTM-PD than in healthy controls (11/150 (7.3%) vs 0/150 (0%), *p* < 0.001). Except for vitamin D, vitamin deficiency status was observed only in patients with NTM-PD. Multiple vitamin deficiencies were only observed in patients with NTM-PD (7.3% of all NTM-PD patients), with combined vitamin A and D deficiency being common. An additional analysis of the association of severe vitamin D deficiency (serum 25(OH)D level < 10 ng/mL) and patients with NTM-PD did not show a significant result. Bivariate logistic regression analysis revealed that serum concentrations of vitamin A, vitamin E, and MMA were related to NTM-PD as shown in Table 2. However, vitamin E or vitamin B_12_ deficiency status was not associated with NTM-PD (Table 2). In an analysis by etiology of NTM-PD, only serum concentrations of vitamin A showed a significant difference (*M. avium*, 1.7 µmol/L; *M. intracellulare*, 1.5 µmol/L; *M. abscessus*, 1.3 µmol/L; and *M. massiliense*, 1.6 µmol/L, *p* = 0.010).

### 3.3. Factors Associated with Vitamin Concentrations of the Study Population

Table 3 shows the correlations among concentrations of vitamins, demographic data, and biochemical results. Positive correlations among vitamins (vitamins A with D, r = 0.200; vitamins D with E, r = 0.238; and vitamins A with E, r = 0.352, *p* < 0.05) were observed in the study population. Homocysteine and MMA were also positively correlated (r = 0.247, *p* < 0.05).

BMI and total cholesterol were positively correlated with vitamin A and/or vitamin E concentrations. The strongest correlation found in the study population was between vitamin E and total cholesterol (r = 0.593, *p* < 0.05). Vitamin A showed positive correlations with total cholesterol (r = 0.314, *p* < 0.05) and BMI (r = 0.367, *p* < 0.05) and a negative correlation with CRP (r = −0.374, *p* < 0.05). Multivariable regression analysis showed that, BMI, albumin, total protein, total cholesterol, and ALT were related to vitamin A. Vitamin D and homocysteine were associated with age, and vitamin E was associated with age, sex, and total cholesterol.

### 3.4. Associations between Vitamin Concentrations and Treatment Outcome of NTM-PD

Twenty-nine patients with NTM-PD did not have an antibiotic treatment record, but 121 patients received antibiotic treatment and regular sputum culture tests. Among these 121 patients, 11 were lost to follow up and 110 patients were finally reviewed for treatment outcome analysis. Seventy-seven patients (70%) achieved sputum culture conversion within 12 months of treatment. Serum vitamin concentrations were increased in the treatment success group but not significantly (Table 4). As shown in Table 4, serum vitamin status, demographic variables, and biochemical indicators did not show significant differences between the sputum culture conversion group and the treatment failure group.

## 4. Discussion

This is the first comprehensive comparative investigation of vitamin status in patients with NTM-PD compared with age- and sex-matched healthy controls. We performed a case-control study to assess the relationship of vitamin status with NTM-PD, and we also studied possible relationships between clinical data, laboratory results, and treatment outcomes with vitamin status in patients with NTM-PD.

Micronutrients such as vitamins can affect several components of innate immunity, and vitamin deficiencies influence host immunity to various infections [29,30]. Deficiencies in vitamins A and D may reduce natural killer cell function [29], and although vitamin E deficiency is rare in healthy subjects, it causes damage to both T cells and B cells [31]. Vitamin B_12_ plays an important role in immune system regulation through effects on cytotoxic cells, and a previous study showed that the percentage of CD4+CD25+ regulatory T cells was lower in vitamin B_12_-deficient babies than in control subjects [32,33]. Absorbing and retaining essential vitamins is important to human health, and a lack of vitamins can cause problems with the immune system.

In this study, serum vitamin A concentrations in patients with NTM-PD were significantly lower than in healthy controls. In a previous study, baseline vitamin A deficiency was associated with a 10-fold increase in risk of TB disease (adjusted odds ratio, 10.53; *p* < 0.001) [8]. Similarly, our study found that serum concentration of vitamin A was associated with an 11-fold increase in risk of NTM-PD, demonstrating that vitamin A deficiency was more common in patients with NTM-PD than in healthy controls. Since vitamin A concentrations are lower in subjects with high serum CRP levels [34], we performed statistical analysis of vitamin A concentrations after excluding subjects with high CRP levels; the results were not significantly different. Recently, Coleman reported that a metabolite of vitamin A (all-*trans* retinoic acid, atRA) promotes macrophage autophagy and reduces bacterial burden in human macrophages infected with *Mycobacterium tuberculosis* [35]. The atRA-induced augmentation of the autophagy pathway was also observed in other respiratory infections. Moreover, Wheelwright showed that vitamin A-triggered anti-*M. tuberculosis* activity requires expression of Niemann-Pick disease type C2 protein [36]. These results indicate that low concentrations of vitamin A may be associated with a compromised ability of the host to inhibit mycobacterial infection. Vitamin A is thought to be important in protection against mycobacterial infection, and understanding this process will be helpful in the treatment of patients [37].

Multiple studies have examined the role of vitamin D in various infectious diseases [38]. In our study, serum vitamin D concentrations did not differ significantly between patients with NTM-PD and healthy controls. Additionally, NTM-PD was not associated with vitamin D deficiency or severe vitamin D deficiency. A similar result was reported in patients with NTM-PD (24.1 ng/mL in NTM-positive patients and 22.8 ng/mL in NTM-negative patients, *p* = 0.21) in a cystic fibrosis population [39]. Contrary to our findings, a lower median concentration of serum 25(OH)D and an association with severe vitamin D deficiency were previously reported in patients with NTM-PD, in a case-control study [40]. The different analytical methods used to measure vitamin D in the two studies may be the cause of the discrepancies, as serum 25(OH)D concentration was measured using an enzyme-linked immunosorbent assay (ELISA) kit (Immundiagnostik AG, Bensheim, Germany) in the previous study and by LC-MS/MS in this study. The 25(OH)D concentration measured by the ELISA was biased −14.2 ± 91.0 nmol/L (bias ± SD) from the LC-MS/MS method, and therefore, the number of patients with vitamin D deficiency could have been overestimated in the previous study [41]. In cases with strong binding of 25(OH)D to vitamin D-binding protein, the total 25(OH)D concentration may not be sufficient to accurately evaluate vitamin D status [42]. We believe that our results may be more accurate because we used the LC-MS/MS method, which is considered the gold standard of vitamin D analysis, and previous studies have shown that this method has good sensitivity and accuracy for quantifying both 25(OH)D2 and 25(OH)D3 [43,44,45,46]. Moreover, a review of factors contributing to susceptibility to NTM-PD found no direct evidence of a role for vitamin D deficiency [47], and Kim reported that a higher level of gene expression for antimicrobial peptide is more likely to be associated with NTM-PD than serum vitamin D status [48]. Several studies have shown an association between vitamin D receptor gene polymorphism and the risk of TB [49,50], but they were not conclusive in regard to NTM-PD [49,51,52]. Unlike in TB, the role of vitamin D in NTM-PD seems to be limited, possibly due to the differences in virulence of the causative organisms and the ensuing immune responses.

We found that serum vitamin E concentrations among patients with NTM-PD were lower than among healthy controls. Although no previous study has evaluated vitamin E in patients with NTM-PD, vitamin E concentrations were reportedly lower in patients with TB than in healthy subjects [6,10,53]. Vitamin E is an efficient antioxidant and plays a role in regulation of the immune system [54,55]. Oxidants play a significant role in lung injury, and a temporary vitamin E deficiency has been reported to induce reversible changes in expression of pro-inflammatory and anti-inflammatory markers [56]. Consumption of vitamin E-selenium supplements has shown potential in reducing reactive oxygen species and increasing antioxidant activities in patients with TB [57].

We used serum MMA and homocysteine as biomarkers of vitamin B_12_ status because they are the most sensitive and specific indicators of functional vitamin B_12_ deficiency [58]. Serum MMA concentrations in patients with NTM-PD were significantly higher than in healthy controls, but homocysteine concentrations did not differ between the two groups. Although both MMA and homocysteine are used to identify vitamin B_12_ deficiency, MMA is known to be a more sensitive and specific biomarker for diagnosis of vitamin B_12_ deficiency [58]. Comparison of genome sequences of *M. tuberculosis* with NTM species (i.e., *M. marinum* and *M. kansasii*) revealed diversity among genes associated with vitamin B_12_-related metabolism [20]. However, measurements of serum MMA showed a similar pattern of higher concentrations in patients with NTM-PD and TB than in healthy controls [6].

In regard to demographic characteristics, patients with NTM-PD had a lower BMI when compared to healthy controls, consistent with previous studies [59,60,61,62]. In addition, positive correlations were found between serum concentrations of vitamin A, D, and E, and BMI. This result could be due to the fat solubility of these vitamins and their known associations with BMI [63]. In our study, total protein and total cholesterol concentrations were also lower in patients with NTM-PD than in healthy controls. Low total cholesterol concentrations in patients with NTM-PD were reported in a previous study [62]. Both reduced synthesis and enhanced metabolism may be the reason for lower total cholesterol concentrations in patients with NTM-PD [64]. In our study, total cholesterol concentration showed the strongest correlation with vitamin E concentration in a correlation analysis between vitamin concentrations and nutritional status-associated parameters. It has been reported that there are significant correlations between serum cholesterol and vitamin E [65]. Vitamin A is another fat-soluble vitamin that correlates with lipids almost as strongly as vitamin E does [66,67].

There was no association of vitamin status with treatment outcome of NTM-PD in this study. To our knowledge, no studies have identified an association between vitamin status and treatment outcome of NTM-PD. However, plasma levels of vitamin A appear to increase following initiation of TB treatment [68]. The association between vitamin D status and TB treatment outcome is inconsistent in previous studies [68,69]. Future well-designed randomized controlled trials among patients with NTM-PD would be helpful to clarify this issue.

Our study had a few limitations. Serum vitamin concentrations obtained with a single measurement may not reflect long-term vitamin status, and we could not obtain data on dietary supplementation of vitamins in the study population. Future well-designed randomized controlled trials are needed to explore whether vitamin status is associated with treatment outcome in NTM-PD.

## 5. Conclusions

In conclusion, we report the first comparison of the status of multiple vitamins in patients with NTM-PD and healthy controls. Our study suggests that altered vitamin status is associated with mycobacterial disease, and that vitamin A might play an important role in NTM-PD. Hence, future well-designed prospective studies with large patient cohorts addressing these issues are needed to clarify the significance of vitamins in NTM-PD.

## Figures and Tables

**Table 1:** General characteristics of the study population.

	NTM Patients (*n* = 150)	Controls (*n* = 150)	*p*-Value
**Demographic characteristics**			
Age, years	59 (52–67) ^a^	58 (53–65)	0.691
Female, N (%)	106 (70.7)	106 (70.7)	1.000
BMI, kg/m^2^	20.7 (19.2–22.3)	23.3 (21.2–24.8)	<0.001
BMI < 18.5 kg/m^2^	25 (16.7)	4 (2.7)	<0.001
BMI ≥ 18.5 kg/m^2^	125 (83.3)	146 (97.3)	
**Serum chemistry results**			
Total protein (g/dL)	7.4 (7.1–7.7)	7.0 (6.8–7.4)	<0.001
Albumin (g/dL)	4.4 (4.3–4.6)	4.3 (4.2–4.5)	0.001
Albumin/globulin ratio	1.5 (1.4–1.7)	1.6 (1.5–1.7)	0.001
CRP (mg/dL)	0.12 (0.05–0.57)	0.04 (0.03–0.07)	<0.001
Total cholesterol (mg/dL)	180.5 (158.8–201.0)	196.5 (178.0–223.0)	<0.001
AST (U/L)	21.0 (18.0–25.0)	21.0 (18.0–25.0)	0.976
ALT (U/L)	17.0 (12.0–21.0)	17.5 (13.8–24.3)	0.165

Abbreviations: NTM, nontuberculous mycobacteria; BMI, body mass index; CRP, C-reactive protein; AST, aspartate aminotransferase; ALT, alanine aminotransferase. ^a^ Results are presented as median (interquartile range) or number (%) with p-values from the Wilcoxon Mann-Whitney test.

**Table 2:** Vitamins and vitamin B_12_ indicators in the study population.

	NTM Patients (*n* = 150)	Controls (*n* = 150)	*p*-Value	Odds Ratio (95% CI)
Serum vitamin concentrations ^a^				
Vitamin A (µmol/L)	1.5 (1.2–1.8)	2.1 (1.8–2.4)	< 0.001	0.091 (0.049–0.170)
Vitamin D (ng/mL)	20.2 (11.9–29.8)	20.3 (14.3–27.6)	0.678	0.995 (0.974–1.017)
Vitamin E (µmol/L)	27.3 (23.3–32.1)	33.1 (27.4–39.6)	< 0.001	0.921 (0.892–0.950)
Homocysteine (µmol/L)	10.2 (8.2–12.4)	9.9 (8.2–11.8)	0.101	1.063 (0.988–1.143)
Methylmalonic acid (nmol/L)	173.8 (135.9–240.6)	132.9 (101.5–180.8)	< 0.001	1.008 (1.005–1.012)
Vitamin deficiency ^b^				
Vitamin A deficiency	11 (7.3%)	0 (0.0%)	0.001	
Vitamin D deficiency	75 (50.0%)	73 (48.7%)	0.908	
Vitamin E deficiency	0 (0.0%)	0 (0.0%)	1.000	
Vitamin B_12_ deficiency	2 (1.3%)	0 (0.0%)	0.498	
Vitamin A and D deficiency	9 (6.0%)	0 (0.0%)	0.004	
Vitamin D and B_12_ deficiency	2 (2.6%)	0 (0.0%)	0.498	

Abbreviations: NTM, nontuberculous mycobacteria; CI, confidence interval. ^a^ Results are presented as median (interquartile range) with *p*-values from logistic regression analysis. ^b^ Results are presented as number (percentage) with *p*-values from the Chi-square test and Fisher’s exact test. No concomitant deficiencies of vitamins A and E, vitamins A and B_12,_ vitamins B_12_ and E, or vitamins D and E were observed.

**Table 3:** Correlations among vitamin status, basal characteristics, and other biochemical test results of the study population ^a^.

	Age	Sex	BMI	Total Protein	Albumin	CRP	Total Cholesterol	AST	ALT
Vitamin A	0.080	−0.112	0.367 ^b^	−0.253 ^b^	0.103	−0.374 ^b^	0.314 ^b^	0.069	0.286 ^b^
Vitamin D	0.187 ^b^	−0.078	0.132 ^b^	−0.095	−0.017	−0.025	0.011	0.101	0.096
Vitamin E	0.154 ^b^	0.176 ^b^	0.141 ^b^	−0.130 ^b^	−0.081	−0.109	0.593 ^b^	0.068	0.105
Homocysteine	0.277 ^b^	−0.277 ^b^	0.032	0.147 ^b^	0.090	0.157 ^b^	0.004	0.054	0.026
Methylmalonic acid	0.114 ^b^	0.038	−0.209 ^b^	0.069	−0.036	0.193 ^b^	−0.081	0.033	−0.067

Abbreviations: BMI, body mass index; CRP, C-reactive protein; AST, aspartate aminotransferase; ALT, alanine aminotransferase. ^a^ Results are presented as Spearman’s correlation coefficient. ^b^ *p*-value < 0.05.

**Table 4:** Serum vitamin status and treatment outcomes in patients with NTM-PD.

	Success (*N* = 77)	Failure (*N* = 33)	*p*-Value
Demographic characteristics			
Age, years	59 (53–67) ^a^	58 (49–70)	0.966
Female, *N* (%)	53 (69%)	23 (70%)	0.929
BMI, kg/m^2^	20.8 (13.9–30.5)	20.3 (19.5–21.2)	0.650
Sputum smear-positive	31 (40%)	20 (61%)	0.051
Cavitary lesion-positive	33 (43%)	14 (42%)	0.967
Serum chemistry results			
Total protein (g/dL)	7.4 (7.2–7.8)	7.6 (7.4–7.9)	0.133
Albumin (g/dL)	4.4 (4.3–4.7)	4.4 (4.2–4.7)	0.613
Albumin/globulin ratio	1.5 (1.4–1.7)	1.5 (1.2–1.6)	0.463
CRP (mg/dL)	0.16 (0.06–0.65)	0.17 (0.07–1.04)	0.511
Total cholesterol (mg/dL)	180 (155–201)	176 (162–192)	0.858
AST (U/L)	22 (18–25)	19 (17–24)	0.114
ALT (U/L))	17 (14–21)	15 (11–19)	0.051
ESR (mm)	37 (22–52)	49 (26–80)	0.082
Serum vitamin concentrations			
Vitamin A (µmol/L)	1.5 (1.2–1.8)	1.3 (1.2–1.6)	0.118
Vitamin D (ng/mL)	20.8 (12.5–29.6)	16.6 (10.2–29.9)	0.459
Vitamin E (µmol/L)	27.1 (23.3–32.6)	26.3 (21.7–29.4)	0.252
Homocysteine (µmol/L)	10.3 (8.4–12.7)	9.6 (7.8–11.7)	0.615
Methylmalonic acid (nmol/L)	173.4 (135.4–244.2)	167.7 (131.3–223.1)	0.148

Abbreviations: NTM, nontuberculous mycobacteria; BMI, body mass index; CRP, C-reactive protein; AST, aspartate aminotransferase; ALT, alanine aminotransferase; ESR, erythrocyte sedimentation rate. ^a^ Results are presented as median (interquartile range) or number (%) with *p*-values from the Wilcoxon Mann-Whitney test.

## References

[1] Prevots D.R., Marras T.K. (2015). Epidemiology of human pulmonary infection with nontuberculous mycobacteria: A review. Clin. Chest Med..

[2] Stout J.E., Koh W.J., Yew W.W. (2016). Update on pulmonary disease due to non-tuberculous mycobacteria. Int. J. Infect. Dis..

[3] Ko R.E., Moon S.M., Ahn S., Jhun B.W., Jeon K., Kwon O.J., Huh H.J., Ki C.S., Lee N.Y., Koh W.J. (2018). Changing epidemiology of nontuberculous mycobacterial lung diseases in a tertiary referral hospital in Korea between 2001 and 2015. J. Korean Med. Sci..

[4] Philley J.V., Griffith D.E. (2015). Treatment of slowly growing mycobacteria. Clin. Chest Med..

[5] Koh W.J., Stout J.E., Yew W.W. (2014). Advances in the management of pulmonary disease due to *Mycobacterium abscessus* complex. Int. J. Tuberc. Lung Dis..

[6] Oh J., Choi R., Park H.D., Lee H., Jeong B.H., Park H.Y., Jeon K., Kwon O.J., Koh W.J., Lee S.Y. (2017). Evaluation of vitamin status in patients with pulmonary tuberculosis. J. Infect..

[7] Pakasi T.A., Karyadi E., Wibowo Y., Simanjuntak Y., Suratih N.M., Salean M., Darmawidjaja N., van der Meer J.W., van der Velden K., Dolmans W.M. (2009). Vitamin A deficiency and other factors associated with severe tuberculosis in Timor and Rote Islands, East Nusa Tenggara Province, Indonesia. Eur. J. Clin. Nutr..

[8] Aibana O., Franke M.F., Huang C.C., Galea J.T., Calderon R., Zhang Z., Becerra M.C., Smith E.R., Ronnenberg A.G., Contreras C. (2017). Impact of Vitamin A and carotenoids on the risk of tuberculosis progression. Clin. Infect. Dis..

[9] Aibana O., Franke M.F., Huang C.C., Galea J.T., Calderon R., Zhang Z., Becerra M.C., Smith E.R., Contreras C., Yataco R. (2018). Vitamin E status is inversely associated with risk of incident tuberculosis disease among household contacts. J. Nutr..

[10] Plit M.L., Theron A.J., Fickl H., van Rensburg C.E., Pendel S., Anderson R. (1998). Influence of antimicrobial chemotherapy and smoking status on the plasma concentrations of vitamin C, vitamin E, beta-carotene, acute phase reactants, iron and lipid peroxides in patients with pulmonary tuberculosis. Int. J. Tuberc. Lung Dis..

[11] Qrafli M., El Kari K., Aguenaou H., Bourkadi J.E., Sadki K., El Mzibri M. (2017). Low plasma vitamin A concentration is associated with tuberculosis in Moroccan population: A preliminary case control study. BMC Res. Notes.

[12] Kim J.H., Park J.S., Cho Y.J., Yoon H.I., Song J.H., Lee C.T., Lee J.H. (2014). Low serum 25-hydroxyvitamin D level: An independent risk factor for tuberculosis?. Clin. Nutr..

[13] Hong J.Y., Kim S.Y., Chung K.S., Kim E.Y., Jung J.Y., Park M.S., Kim Y.S., Kim S.K., Chang J., Kang Y.A. (2014). Association between vitamin D deficiency and tuberculosis in a Korean population. Int. J. Tuberc. Lung Dis..

[14] Ustianowski A., Shaffer R., Collin S., Wilkinson R.J., Davidson R.N. (2005). Prevalence and associations of vitamin D deficiency in foreign-born persons with tuberculosis in London. J. Infect..

[15] Wejse C., Olesen R., Rabna P., Kaestel P., Gustafson P., Aaby P., Andersen P.L., Glerup H., Sodemann M. (2007). Serum 25-hydroxyvitamin D in a West African population of tuberculosis patients and unmatched healthy controls. Am. J. Clin. Nutr..

[16] Ho-Pham L.T., Nguyen N.D., Nguyen T.T., Nguyen D.H., Bui P.K., Nguyen V.N., Nguyen T.V. (2010). Association between vitamin D insufficiency and tuberculosis in a Vietnamese population. BMC Infect. Dis..

[17] Mastala Y., Nyangulu P., Banda R.V., Mhemedi B., White S.A., Allain T.J. (2013). Vitamin D deficiency in medical patients at a central hospital in Malawi: A comparison with TB patients from a previous study. PLoS ONE.

[18] Venturini E., Facchini L., Martinez-Alier N., Novelli V., Galli L., de Martino M., Chiappini E. (2014). Vitamin D and tuberculosis: A multicenter study in children. BMC Infect. Dis..

[19] Gopinath K., Venclovas C., Ioerger T.R., Sacchettini J.C., McKinney J.D., Mizrahi V., Warner D.F. (2013). A vitamin B_12_ transporter in *Mycobacterium tuberculosis*. Open Biol..

[20] Young D.B., Comas I., de Carvalho L.P.S. (2015). Phylogenetic analysis of vitamin B_12_-related metabolism in *Mycobacterium tuberculosis*. Front. Mol. Biosci..

[21] Griffith D.E., Aksamit T., Brown-Elliott B.A., Catanzaro A., Daley C., Gordin F., Holland S.M., Horsburgh R., Huitt G., Iademarco M.F. (2007). An official ATS/IDSA statement: Diagnosis, treatment, and prevention of nontuberculous mycobacterial diseases. Am. J. Respir. Crit. Care Med..

[22] Koh W.J., Moon S.M., Kim S.Y., Woo M.A., Kim S., Jhun B.W., Park H.Y., Jeon K., Huh H.J., Ki C.S. (2017). Outcomes of *Mycobacterium avium* complex lung disease based on clinical phenotype. Eur. Respir. J..

[23] Koh W.J., Jeong B.H., Kim S.Y., Jeon K., Park K.U., Jhun B.W., Lee H., Park H.Y., Kim D.H., Huh H.J. (2017). Mycobacterial characteristics and treatment outcomes in *Mycobacterium abscessus* lung disease. Clin. Infect. Dis..

[24] Koh W.J., Jeong B.H., Jeon K., Kim S.Y., Park K.U., Park H.Y., Huh H.J., Ki C.S., Lee N.Y., Lee S.H. (2016). Oral macrolide therapy following short-term combination antibiotic treatment for *Mycobacterium massiliense* lung disease. Chest.

[25] Who Expert Consultation (2004). Appropriate body-mass index for Asian populations and its implications for policy and intervention strategies. Lancet.

[26] Van Ingen J., Aksamit T., Andrejak C., Bottger E.C., Cambau E., Daley C.L., Griffith D.E., Guglielmetti L., Holland S.M., Huitt G.A. (2018). Treatment outcome definitions in nontuberculous mycobacterial pulmonary disease: An NTM-NET consensus statement. Eur. Respir. J..

[27] Rwangabwoba J.M., Fischman H., Semba R.D. (1998). Serum vitamin A levels during tuberculosis and human immunodeficiency virus infection. Int. J. Tuberc. Lung Dis..

[28] De Pee S., Dary O. (2002). Biochemical indicators of vitamin A deficiency: Serum retinol and serum retinol binding protein. J. Nutr..

[29] Erickson K.L., Medina E.A., Hubbard N.E. (2000). Micronutrients and innate immunity. J. Infect. Dis..

[30] Katona P., Katona-Apte J. (2008). The interaction between nutrition and infection. Clin. Infect. Dis..

[31] Pekmezci D. (2011). Vitamin E and immunity. Vitam. Horm..

[32] Tamura J., Kubota K., Murakami H., Sawamura M., Matsushima T., Tamura T., Saitoh T., Kurabayshi H., Naruse T. (1999). Immunomodulation by vitamin B12: Augmentation of CD8+ T lymphocytes and natural killer (NK) cell activity in vitamin B12-deficient patients by methyl-B12 treatment. Clin. Exp. Immunol..

[33] Boran P., Yildirim S., Karakoc-Aydiner E., Ogulur I., Ozen A., Haklar G., Koc A., Akkoc T., Barlan I. Vitamin B12 deficiency among asymptomatic healthy infants: Its impact on the immune system. https://europepmc.org/abstract/med/26763692.

[34] Thurnham D.I., McCabe G.P., Northrop-Clewes C.A., Nestel P. (2003). Effects of subclinical infection on plasma retinol concentrations and assessment of prevalence of vitamin A deficiency: Meta-analysis. Lancet.

[35] Coleman M.M., Basdeo S.A., Coleman A.M., Cheallaigh C.N., Peral de Castro C., McLaughlin A.M., Dunne P.J., Harris J., Keane J. (2018). All-trans retinoic acid augments autophagy during intracellular bacterial infection. Am. J. Respir. Cell Mol. Biol..

[36] Wheelwright M., Kim E.W., Inkeles M.S., De Leon A., Pellegrini M., Krutzik S.R., Liu P.T. (2014). All-trans retinoic acid-triggered antimicrobial activity against *Mycobacterium tuberculosis* is dependent on NPC2. J. Immunol..

[37] Aggarwal S., DeBerry J. (2018). Can a vitamin a day keep tuberculosis away?. Am. J. Respir. Cell Mol. Biol..

[38] Yamshchikov A.V., Desai N.S., Blumberg H.M., Ziegler T.R., Tangpricha V. (2009). Vitamin D for treatment and prevention of infectious diseases: A systematic review of randomized controlled trials. Endocr. Pract..

[39] Bar-On O., Mussaffi H., Mei-Zahav M., Prais D., Steuer G., Stafler P., Hananya S., Blau H. (2015). Increasing nontuberculous mycobacteria infection in cystic fibrosis. J. Cyst. Fibros..

[40] Jeon K., Kim S.Y., Jeong B.H., Chang B., Shin S.J., Koh W.J. (2013). Severe vitamin D deficiency is associated with non-tuberculous mycobacterial lung disease: A case-control study. Respirology.

[41] He C.S., Gleeson M., Fraser W.D. (2013). Measurement of circulating 25-hydroxy vitamin d using three commercial enzyme-linked immunosorbent assay kits with comparison to liquid chromatography: Tandem mass spectrometry method. ISRN Nutr..

[42] Kim H.J., Ji M., Song J., Moon H.W., Hur M., Yun Y.M. (2017). Clinical utility of measurement of vitamin D-binding protein and calculation of bioavailable vitamin D in assessment of vitamin D status. Ann. Lab. Med..

[43] Le Goff C., Cavalier E., Souberbielle J.C., Gonzalez-Antuna A., Delvin E. (2015). Measurement of circulating 25-hydroxyvitamin D: A historical review. Pract. Lab. Med..

[44] Arneson W.L., Arneson D.L. (2013). Current methods for routine clinical laboratory testing of vitamin D levels. Lab. Med..

[45] Koivula M.K., Matinlassi N., Laitinen P., Risteli J. (2013). Four automated 25-OH total vitamin D immunoassays and commercial liquid chromatography tandem-mass spectrometry in Finnish population. Clin. Lab..

[46] Kwak H.-S., Chung H.-J., Cho D.-H., Park M.-H., Ku E.-S., Park E.J., Oh H.J. (2015). Efficacy of the measurement of 25-hydroxyvitamin D2 and D3 levels by using PerkinElmer liquid chromatography-tandem mass spectrometry vitamin D kit compared with DiaSorin radioimmunoassay kit and Elecsys vitamin D total assay. Ann. Lab. Med..

[47] Sexton P., Harrison A.C. (2008). Susceptibility to nontuberculous mycobacterial lung disease. Eur. Respir. J..

[48] Kim S.Y., Chang B., Jeong B.H., Park H.Y., Jeon K., Shin S.J., Koh W.J. (2016). Implication of vitamin D-associated factors in patients with non-tuberculous mycobacterial lung disease. Int. J. Tuberc. Lung Dis..

[49] Lewis S.J., Baker I., Davey Smith G. (2005). Meta-analysis of vitamin D receptor polymorphisms and pulmonary tuberculosis risk. Int. J. Tuberc. Lung Dis..

[50] Gao L., Tao Y., Zhang L., Jin Q. (2010). Vitamin D receptor genetic polymorphisms and tuberculosis: Updated systematic review and meta-analysis. Int. J. Tuberc. Lung Dis..

[51] Gelder C.M., Hart K.W., Williams O.M., Lyons E., Welsh K.I., Campbell I.A., Marshall S.E. (2000). Vitamin D receptor gene polymorphisms and susceptibility to *Mycobacterium malmoense* pulmonary disease. J. Infect. Dis..

[52] Park S., Kim E.J., Lee S.H., Suh G.Y., Chung M.P., Kim H., Kwon O.J., Koh W.J. (2008). Vitamin D-receptor polymorphisms and non-tuberculous mycobacterial lung disease in Korean patients. Int. J. Tuberc. Lung Dis..

[53] Madebo T., Lindtjorn B., Aukrust P., Berge R.K. (2003). Circulating antioxidants and lipid peroxidation products in untreated tuberculosis patients in Ethiopia. Am. J. Clin. Nutr..

[54] Traber M.G., Atkinson J. (2007). Vitamin E, antioxidant and nothing more. Free Radic. Biol. Med..

[55] Lee C.Y., Man-Fan Wan J. (2000). Vitamin E supplementation improves cell-mediated immunity and oxidative stress of Asian men and women. J. Nutr..

[56] Kolleck I., Sinha P., Rustow B. (2002). Vitamin E as an antioxidant of the lung: Mechanisms of vitamin E delivery to alveolar type II cells. Am. J. Respir. Crit. Care Med..

[57] Seyedrezazadeh E., Ostadrahimi A., Mahboob S., Assadi Y., Ghaemmagami J., Pourmogaddam M. (2008). Effect of vitamin E and selenium supplementation on oxidative stress status in pulmonary tuberculosis patients. Respirology.

[58] Stabler S.P. (2013). Vitamin B12 deficiency. N. Engl. J. Med..

[59] Chan E.D., Iseman M.D. (2010). Slender, older women appear to be more susceptible to nontuberculous mycobacterial lung disease. Gend. Med..

[60] Wakamatsu K., Nagata N., Maki S., Omori H., Kumazoe H., Ueno K., Matsunaga Y., Hara M., Takakura K., Fukumoto N. (2015). Patients with MAC lung disease have a low visceral fat area and low nutrient intake. Pulm. Med..

[61] Ikegame S., Maki S., Wakamatsu K., Nagata N., Kumazoe H., Fujita M., Nakanishi Y., Kawasaki M., Kajiki A. (2011). Nutritional assessment in patients with pulmonary nontuberculous mycobacteriosis. Intern. Med..

[62] Hong J.Y., Yang G.E., Ko Y., Park Y.B., Sim Y.S., Park S.H., Lee C.Y., Jung K.-S., Lee M.G. (2016). Changes in cholesterol level correlate with the course of pulmonary nontuberculous mycobacterial disease. J. Thorac. Dis..

[63] Burtis C.A., Ashwood E.R., Bruns D.E. (2012). Tietz Textbook of Clinical Chemistry and Molecular Diagnostics-e-Book.

[64] Vyroubal P., Chiarla C., Giovannini I., Hyspler R., Ticha A., Hrnciarikova D., Zadak Z. (2008). Hypocholesterolemia in clinically serious conditions—Review. Biomed. Pap. Med. Fac. Univ. Palacky Olomouc Czechoslov. Repub..

[65] Cham B.E., Smith J.L., Colquhoun D.M. (1998). Correlations between cholesterol, vitamin E, and vitamin K1 in serum: Paradoxical relationships to established epidemiological risk factors for cardiovascular disease. Clin. Chem..

[66] Gey K.F., Puska P. (1989). Plasma vitamins E and A inversely correlated to mortality from ischemic heart disease in cross-cultural epidemiology. Ann. N. Y. Acad. Sci..

[67] Jordan P., Brubacher D., Moser U., Stahelin H.B., Gey K.F. (1995). Vitamin E and vitamin A concentrations in plasma adjusted for cholesterol and triglycerides by multiple regression. Clin. Chem..

[68] Grobler L., Nagpal S., Sudarsanam T.D., Sinclair D. (2016). Nutritional supplements for people being treated for active tuberculosis. Cochrane Database Syst. Rev..

[69] Mehta S., Mugusi F.M., Bosch R.J., Aboud S., Urassa W., Villamor E., Fawzi W.W. (2013). Vitamin D status and TB treatment outcomes in adult patients in Tanzania: A cohort study. BMJ Open.

